# Skin Cancer Prevention Coverage in Popular US Women’s Health and Fitness Magazines: An Analysis of Advertisements and Articles

**DOI:** 10.5539/gjhs.v6n4p42

**Published:** 2014-04-02

**Authors:** Corey Hannah Basch, Danna Ethan, Grace Clarke Hillyer, Alyssa Berdnik

**Affiliations:** 1 William Paterson University, Wayne, United States; 2 Lehman College, City University of New York, New York, United States; 3 Columbia University, Mailman School of Public Health, New York, United States

**Keywords:** skin cancer, prevention, women’s magazines, advertisements, articles

## Abstract

The desire to be tan is a phenomenon that public health researchers have investigated, as exposure to UV radiation increases the chances of developing skin cancer. Media messages in women’s magazines have been shown to contribute to this problem. Much less is known about the prevalence of skin cancer prevention messages in these magazines. This study’s aim was to identify the number and type of articles and advertised products devoted to skin health (sun protection and skin cancer prevention in particular) within five popular U.S. greater than women’s health and fitness magazines. We analyzed articles and advertisements over seven months of issues of the following popular women’s health and fitness magazines: Fitness, Health, Self, Shape, and Women’s Health, March 2013 through September 2013. Overall, 31 issues of the five magazines with a total of 780 articles and 1,986 advertisements were analyzed. Of the 780 articles, a mere 2.9% (n=23) were devoted to skin. Of the 258 skin product advertisements, less than 20% of the products contained sun protection factor (SPF). These findings suggest that women’s health and fitness magazines can improve their efforts in informing women of skin cancer risks and preventive measures to minimize these risks. The role of these magazines in building health literacy among their readers is also discussed.

## 1. Introduction

The most common form of cancer among Americans is skin cancer (Centers for Disease Control and Prevention [CDC], 2013). An estimated 3.5 million cases of non-melanoma skin cancer are diagnosed each year with one in five Americans developing the disease in their lifetime (American Academy of Dermatology [AAD], 2013). Incidence of melanoma, the deadliest form of skin cancer, has increased steadily in the last thirty years, particularly among Caucasian women under 44 years old, a statistic that may be related to recent indoor tanning trends (AAD, 2013; Little & Eide, 2012). Indoor tanning practices and sun exposure are major risk factors for skin cancer because of exposure to ultraviolet (UV) light. When UV rays reach the skin’s inner layer, the skin produces more melanin and moves towards the outer layers of the skin thereby producing a “tan” (American Cancer Society, 2013). This tan is a response to injury as the skin cells are signaling their damage by the UV rays by producing more pigment.

The desire to be tan is a phenomenon that public health researchers have investigated. Findings from several studies suggest that the desire to look tan stems from the belief that it enhances appearance (Cafri, Thompson, Jacobsen, & Hillhoouse, 2009; Peacey, Steptoe, Sanderman, & Wardle, 2006; Lamanna, 2004; Dennis, Lowe, & Snetselaar, 2009). Other studies indicate that use of skin products with sun protection factor (SPF) is not desirable, and therefore risk reduction does not occur (Turrisi, Hillhouse, Gebert, & Grimes, 1999; C.H. Basch, Hillyer, C.E. Basch, & Neugut, 2012). Limited knowledge and negative attitudes about sun safety are also cited as reasons for increased risk (AAD, 2013; Diepgen & Mahler, 2002; Lamanna, 2004).

Health and fitness magazines are a popular medium for increasing exposure to health-related content. These magazines generally include articles on such topics as skin health, nutrition, exercise, sexual health, stress management, disease prevention, among others. Read monthly by millions of women of varied ages, these magazines also contain articles, editorials and advertisements that, together, provide a context through which both health and risk behaviors are encouraged (Cho, Hall, Kosmoski, Fox, & Mastin, 2010).

Although a body of research has examined tanning-related media messages in magazines, little attention has been paid to skin protection content in articles and advertisements for products to reduce risk in magazines and no studies have assessed the prevalence of protection/risk reduction messages in popular women’s health and fitness magazines. We contend that magazine articles and advertisements can be an important and accurate source of information for readers on skin cancer prevention and sun protection thereby enhancing readers’ health literacy in this area. Considered an important health promotion mechanism that is underutilized by the public-at-large, health literacy is expressed when an individual identifies and uses health information to make decisions that support health (CDC, 2011). There are multiple channels for imparting health information including doctors’ offices, retail venues, community agencies, and various forms of media (CDC, 2011).

Women’s health and fitness magazines can be considered a channel for information that readers may potentially use to make informed, health-related decisions. To assess the extent to which this magazine genre includes information on skin health, our study’s aim, therefore, was to identify the number and type of articles and advertised products devoted to skin health (sun protection and skin cancer prevention in particular) within five popular women’s health and fitness magazines. For the advertisements, we also aimed to assess 1) the type of product displayed (sun block, sunless tanner, other skin products), and 2) presence and amount of sun protection factor (SPF) in the products advertised.

## 2. Materials and Methods

This study entailed an analysis of seven months of issues of the popular U.S. women’s health and fitness magazines, *Fitness, Health, Self, Shape, and Women’s Health*, covering March 2013 through September 2013. Given the various genres of women’s magazines and the possible differences in products advertised, we selected these magazines with an emphasis on health and well-being as opposed to other contemporary women’s magazines with a focus on beauty and fashion (Dixon, Warne, Scully, Wakefield, & Dobbinson, 2011). The months were selected based on the premise that these issues would be most likely to cover information related to skin protection as they are months when the weather is warmer. It should be noted that one magazine, *Fitness*, is released bi-monthly. These magazines were selected for their expansive reach and their focus on women within the age range and racial background for which melanoma is increasing (Little & Eide, 2012; Women’s Health, 2014; Audit Bureau of Circulations, 2010). The combined readership for these magazines is 36,300,000 with a mean age of 43 years among readers across these selected magazines (MRI+, 2013). In addition, most popular women’s health and fitness magazines are geared towards a Caucasian readership and feature predominantly white models. This population is important, due to their increased risk of developing melanoma.

A coding sheet was adapted based on a prior study that analyzed articles and advertisements related to skin for a different population (Basch et al., 2013). Hard copies of all magazines were obtained and utilized. Articles were identified from the table of contents. Content was assessed and recorded as related to skin (sun protection/risk reduction, skin cancer, beauty or other) or health (behavioral, chronic disease/cancer prevention, emotional health/stress reduction, exercise, nutrition, reproductive health/sex, and other). These themes were identified as emergent when the coding sheet was being developed. We considered all advertisements excluding the front cover, tear-out promotions, and products promoted by the magazine (such as “staff picks”) that were clearly not paid advertisements. The content of advertisements was similarly grouped as skin-related and health-related as well as an “other” category. Skin-related advertisements were further grouped as sunless tanners, sun block of varying levels of SPF, skin products such as moisturizers with and without SPF, and other. Birth control, adult health, and miscellaneous health advertisements were grouped as health-related. Further, when sun block advertisements were identified, a content analysis of the advertisement was conducted noting and categorizing underlying themes. These themes included sun protection, relaxation, sex appeal, enjoyment/fun with friends, beauty, skin protection, and special ingredient/texture and feel/benefit to the skin. It is important to note that products falling into multiple categories were not encountered. Intra-rater reliability was assessed using Cohen’s kappa for a random 20% sample of the magazines and found to be excellent (k = 0.86). Frequency distributions were calculated and the range of items per magazine issue was assessed. This study was determined not to be human subjects research by the Institutional Review Boards at William Paterson University, Lehman College, and Columbia University.

## 3. Results

We analyzed 31 issues of the five popular U.S. women’s health and fitness magazines with a total of 780 articles and 1,986 advertisements. Total articles and advertisements by type are indicated in Table 1.

**Table 1:** Total articles and advertisements by type in Fitness, Health, Self, Shape, and Women’s Health, March 2013 through September 2013 (n = 31)

	N (%)	Range per magazine issue
**Articles**	780	15-39
Skin^1^	23 (2.9)	0-2
Sun protection/risk reduction	15 (31.9)	0-1
Skin cancer	6 (12.8)	0-1
Beauty-related	13 (25.5)	0-1
Other	13 (25.5)	0-1
Health	757 (97.1)	13-38
Behavioral	21 (2.8)	0-3
Chronic disease/cancer prevention	6 (0.8)	0-1
Emotional health/stress reduction	42 (5.5)	0-3
Exercise	120 (15.9)	1-8
Nutrition	168 (22.2)	3-8
Reproductive health/sex	30 (4.0)	0-4
Other	370 (48.8)	4-22
**Advertisements**	1986	0-26
Skin	258 (13.0)	0-11
Sunless tanners	11 (4.3)	0-2
Sun block^2^	30 (11.6)	0-6
15 SPF	9 (16.4)	0-1
30 SPF	23 (41.8)	0-4
>30 SPF	23 (41.8)	0-5
Skin products with SPF	50 (19.4)	0-3
Skin products without SPF	52 (20.2)	0-3
Other	115 (44.6)	0-11
Health	348 (17.5)	0-16
Birth control	21 (6.0)	0-2
Adult health	141 (40.5)	1-11
Miscellaneous health	186 (53.4)	1-16
Other	1380 (69.5)	0-26

*Note:* SPF = sun protection factor.

1 Some articles had more than one theme; total number of skin article themes, n = 47 (range 0-3).

2 Some advertisements portrayed more than one SPF level of the sun block product; total number of products displayed, n = 55 (range 0-5).

Of the 780 articles, a mere 2.9% (n=23) were devoted to skin. Of these, the majority of articles discussed beauty and other aspects of skin care (51%), while nearly one-third were devoted to risk reduction and 12.8% to skin cancer prevention. In terms of other articles related to health, the most commonly cited material focused on exercise (n= 120; 15.9%) and nutrition (n=168; 22.2%). Of the 1,986 advertisements present, 258 (13.0%) were related to skin. Similar numbers of advertisements were devoted to skin products without SPF (n = 52; 20.2%) and with SPF (n =50; 19.4%). We identified 30 (11.6%) advertisements for sun block, the majority of which had an SPF of 30 or higher. The most common theme identified was sun protection, followed by themes related to special ingredients, texture, and benefit to skin (Table 2).

**Table 2:** Sunblock advertisement (n = 30) analysis by theme in *Fitness, Health, Self, Shape, and Women’s Health*, March 2013 through September 2013 (n = 31)

	N (%)	Range per magazine issue
**Theme**	49	0-2
Sun protection^1^	16 (32.6)	0-2
Relaxation	3 (6.1)	0-1
Sex appeal	4 (8.2)	0-1
Enjoyment/fun with friends	1 (2.0)	0-1
Beauty	5 (10.2)	0-1
Skin protection	8 (16.3)	0-2
Special ingredient/texture & feel/benefit to skin	12 (24.5)	0-2

1 Sun protection theme includes depiction of protective clothing, hats, eyewear, and shade.

## 4. Discussion

This study is novel in that it is the first to explore advertisements and articles on skin protection and skin cancer risk reduction in health and fitness magazines for women. We found that only a small fraction of the hundreds of articles that appeared in the spring and summer issues of the most popular women’s health and fitness magazines in 2013 were devoted to skin and, of those, about one-third addressed skin cancer and risk reduction. Advertisements featuring products to protect the skin from the deleterious effects of the sun like sunless tanners, sun block with SPF ≥30, and skin products such as moisturizers with SPF were represented equally as often as those without SPF. Themes most commonly employed to promote these products focused on the ability of the product to protect one’s skin from harmful UV rays and the special ingredients to accomplish this aim while enhancing the skin in some way.

Sun protection factor (SPF), measures a skin product’s capacity to prevent UVB rays from damaging the skin. The higher the SPF, the longer the skin is protected (Skin Cancer Foundation, 2014). Of the skin products advertised in our sample (excluding sun block and sunless tanners), many did not contain SPF. It is recommended that use of skin care products that contain an SPF is sufficient in protecting skin for daily activities with limited sun exposure. Daily use of a skin care product with SPF is a relatively simple measure that women can take to protect their skin from the sun and reduce skin cancer risk (Skin Cancer Foundation, 2014). Additional advertisements of these products in health and fitness magazines can reinforce this health message. Encouragingly, the majority of the advertisements for sun block promoted products with 30 SPF or greater. That so few of the articles in the 31 issues of the five most popular women’s health and fitness magazines address sun protection and risk reduction is of particular concern given that we examined only issues from the spring and summer months. In the summer months, time spent outdoors increases and the need for sun protection is greater.

Fitness and health-focused magazines can contribute more significantly to raising awareness, promoting intentions, and encouraging health-related behaviors that reduce the risk of skin cancer among their readership. A body of research suggests that perception, motives, mood, and behaviors of adult and teenage females can be influenced by reading women’s magazines (Grabe, Ward, & Hyde, 2008; Birkeland et al., 2005). For instance, a study of female college students found that greater levels of mood disturbance and body dissatisfaction were produced by advertisements featuring models than by those featuring products (Birkeland et al., 2005). Findings from a meta-analysis on the role of media in women’s concerns about body image suggested that exposure to magazines and other forms of media were correlated with a greater investment in appearance (Grabe et al., 2008).

Magazines have also been shown to influence women’s attitudes about their skin. A study of Caucasian college women found that those who paid close attention to the tanness of models in health and beauty magazines positively associated those models with being “fit.” In addition, those with positive attitudes towards tanning expressed a belief that tanned women in magazines are “fashionable” (Cho, Lee, & Wilson, 2010). An Australian study found that women’s exposure to popular women’s magazines resulted in an increased chance that the reader would attempt to tan (Dixon et al., 2011). Given this noted influence on women’s intentions, attitudes, and behaviors, health and fitness magazines, read by millions of American women, the majority of which are Caucasian (Wasylkiw, Emms, Meuse, & Poirier, 2009; Duerksen et al., 2005), can serve as an important medium for communicating health messages that are accurate and address relevant health needs of their target population. Findings from a 2009 study of skin cancer beliefs, sun protection behavior and exposure to mass media (Hay et al., 2009) suggest that despite the availability of the internet, print media including magazines is a well-utilized channel for dissemination of health and skin cancer information. The study also found that non-Hispanic white women were exposed to health information in magazines more than women of other ethnicities which points to the importance of using this medium to develop targeted skin health messages.

Consumers are now able to access health information through a variety of mechanisms and rely heavily on what is available to make decisions about their health (Wasylkiw et al., 2009). This can be an overwhelming task even for those who are adept at identifying reliable sources. In addition, interpreting health information is undertaken by those who may not engage frequently with the healthcare system (Peerson & Saunders, 2009). In light of this, magazines of this genre can focus more heavily on providing health-promoting messages through their articles – and advertisements – with the aim of educating women and raising their awareness to help them make better decisions about their health. In so doing, magazines would be supporting women’s health literacy which is defined as the capacity to obtain, understand and use health information and services to make informed decisions and has been deemed a “prescription to end confusion” in a report by the National Research Council (CDC, 2011; Wasylkiw et al., 2009).

Health literacy involves not only knowledge about health topics but also skills such as correctly reading a nutrition label, interpreting blood sugar levels, choosing an appropriate health care plan, or identifying abnormal changes in one’s skin (Duerksen et al., 2005). Both health knowledge and skills related to skin cancer prevention can be addressed more readily in health and fitness magazine articles – particularly during spring and summer months when presumably more readers are spending time outdoors. Even advertisers for skin products can be encouraged to promote health literacy skills through their ads without compromising their end goal. For instance, sunscreen advertisements can provide information on how much sunscreen to use and how often to reapply. Advertisements for skin care products with SPF can reinforce the importance of daily use.

This study is limited in that the results are based on a sample of issues from only spring and summer months from a single year, and our sample size was small. Bias introduced by subjective grading of article and advertisement categorization, as well as theme coding of advertisements, was minimized by excellent intra-rater reliability. This study addresses a gap in the literature related to the prevalence of skin cancer prevention messages in popular U.S. women’s health and fitness magazines by examining the content of five magazines with a combined readership of more than 36 million women, many who are most vulnerable to skin cancer. Our findings suggest that health and fitness magazines, through building health literacy, can play a greater role in helping women make more informed decisions about skin cancer prevention. Further research is necessary to assess potential effects of advertised skin products and skin health articles on intention to use products and/or engage in behaviors that promote skin protection.

## References

[ref1] American Academy of Dermatology (2014). Suntelligence Survey. http://www.aad..org/suntelligence/quiz.aspx.

[ref2] American Academy of Dermatology (2014). Skin cancer. http://www.aad.org/media-resources/stats-and-facts/conditions/skin-cancer.

[ref3] American Cancer Society (2013). Skin cancer.

[ref4] Audit Bureau of Circulations (2010). Health.

[ref5] Basch C. H, Hillyer G. C, Basch C. E, Neugut A. I (2012). Improving understanding about tanning behaviors in college students: A pilot study. The Journal of American College Health.

[ref6] Basch C. H, Hillyer G. C, Basch C. E (2013). Descriptive analysis of articles and advertisements pertaining to skin care and skin cancer prevention in two popular parenting magazines 2000-2010. Preventing Chronic Disease: Public Health Research, Practice and Policy.

[ref7] Birkeland R, Thompson J. K, Herbozo S, Roehrig M, Cafri G, van den Berg P (2005). Media exposure, mood, and body image dissatisfaction: An experimental test of person versus product priming. Body Image.

[ref8] Cafri G, Thompson J.K, Jacobsen P.B, Hillhoouse J (2009). Investigating the role of appearance-based factors in predicting sunbathing and tanning salon use. Journal of Behavioral Medicine.

[ref9] Cartmel B, Ferrucci L. M, Spain P, Bale A. E, Pagoto S. L, Laffell D. J, Mayne S. T (2013). Indoor tanning and tanning dependence in young people after a diagnosis of basal cell carcinoma. JAMA Dermatology.

[ref10] Centers for Disease Control and Prevention (2013a). Basic information about skin cancer.

[ref11] Centers for Disease Control and Prevention (2013b). Skin cancer prevention.

[ref12] Centers for Disease Control and Prevention (2011c). Learn about health literacy.

[ref13] Cho H, Lee S, Wilson K (2010a). Magazine exposure, tanned women stereotypes, and tanning attitudes. Body Image.

[ref14] Cho H, Hall J. G, Kosmoski C, Fox R. L, Mastin T (2010b). Tanning, skin cancer risk, and prevention: A content analysis of eight popular magazines that target female readers 1997–2006. Health Communication.

[ref15] Dennis L. K, Lowe J. B, Snetselaar L. G (2009). Tanning behavior among young frequent tanners is related to attitudes and not lack of knowledge about the dangers. Health Education Journal.

[ref16] Diepgen T. L, Mahler V (2002). The epidemiology of skin cancer. British Journal of Dermatology.

[ref17] Dixon H. G, Warne C. D, Scully M. L, Wakefield M. A, Dobbinson S. J (2011). Does the portrayal of tanning in Australian women’s magazines relate to real women’s tanning beliefs and behavior?. Health education & behavior: the official publication of the Society for Public Health Education.

[ref18] Duerksen S. C, Mikail A, Tom L, Patton A, Lopez J, Amador X, Sadler G. R (2005). Health disparities and advertising content of women’s magazines: a cross-sectional study. BioMed Central Public Health.

[ref19] Grabe S, Ward L. M, Hyde J. S (2008). The role of the media in body image concerns among women: A meta-analysis of experimental and correlational studies. Psychological Bulletin.

[ref20] Hay J, Coups E. J, Ford J, DiBonaventura M (2009). Exposure to mass media health information, skin cancer beliefs, and sun protection behaviors in a United States probability sample. Journal of the American Academy of Dermatology.

[ref21] Heckman C.J, Cohen-Filipic J, Darlow S, Kloss J.D, Manne S.L, Munshi T (2014). Psychiatric and addictive symptoms of young adult female indoor tanners. American Journal of Health Promotion.

[ref22] Lamanna L. M (2004). College students’ knowledge and attitudes about cancer and perceived risks of developing skin cancer. Dermatology nursing/Dermatology Nurses’Association.

[ref23] Little E. G, Eide M. J (2012). Update on the current state of melanoma incidence. Dermatological Clinics.

[ref24] MRI+ (2013). http://www.mriplus.com/publications/searchresults.aspx.

[ref25] National Research Council (2004). Health Literacy: A Prescription to End Confusion.

[ref26] Peacey V, Steptoe A, Sanderman R, Wardle J (2006). Ten-year changes in sun protection behaviors and beliefs of young adults in 13 European countries. Preventive Medicine.

[ref27] Peerson A, Saunders M (2009). Health literacy revisited: What do we mean and why does it matter?. Health Promotion International.

[ref28] Petit A, Lejoyeux M, Reynaud M, Karila L (2013). Excessive indoor tanning as a behavioral addiction: A literature review. Current Pharmaceutical Design.

[ref29] Skin Cancer Foundation (2014a). Prevention Guidelines. http://www.skincancer.org/prevention/sun-protection/prevention-guidelines.

[ref30] Skin Cancer Foundation (2014b). Sunscreens explained. http://www.skincancer.org/prevention/sun-protection/sunscreen/sunscreens-explained.

[ref31] Turrisi R, Hillhouse J, Gebert C, Grimes J (1999). Examination of cognitive variables relevant to sunscreen use. Journal of Behavioral Medicine.

[ref32] U.S. Department of Health and Human Services Office of Disease Prevention and Health Promotion Quick guide to health literacy. http://www.health.gov/communication/literacy/quickguide/factsbasic.htm.

[ref33] Wasylkiw L, Emms A. A, Meuse R, Poirier K. F (2009). Are all models created equal? A content analysis of women in advertisements of fitness versus fashion magazines. Body Image.

[ref34] Women’s Health (2014). Women’s health media kit 2014 circulation summary. http://www.womenshealthmag.com/files/mediakit/WH-MediaKit-CircSummary.pdf.

